# Multilevel and general linear modeling of weather and time effects on the emotional and behavioral states of children with profound intellectual and multiple disabilities

**DOI:** 10.3389/fpsyt.2023.1235582

**Published:** 2024-01-05

**Authors:** Von Ralph Dane Marquez Herbuela, Tomonori Karita, Akihiro Toya, Yoshiya Furukawa, Shuichiro Senba, Eiko Onishi, Tatsuo Saeki

**Affiliations:** ^1^Center for Inclusive Education, Faculty of Education, Ehime University, Ehime, Japan; ^2^Graduate School of Humanities and Social Sciences, Hiroshima University, Higashihiroshima, Japan; ^3^Faculty of Humanities, Fukuoka University, Fukuoka, Japan; ^4^DigitalPia Co., Ltd., Ehime, Japan

**Keywords:** intellectual disability, behavioral measures, emotional states, environmental influences, weather indices, seasonal variations, time, multilevel modeling

## Abstract

**Introduction:**

Eliciting the emotional and behavioral states of children with severe or profound intellectual disabilities (IDs) and profound intellectual and multiple disabilities (PIMD) due to their complex and atypical developmental trajectories has become increasingly elusive. It is evident that the environment, influenced by weather conditions and time of the day, plays a pivotal role in molding children’s behaviors, emotions, and interactions. This underscores the significance of the environment as a critical factor in exploring the communication dynamics of children with PIMD/IDs.

**Methods:**

Over five months during fall and winter seasons, we conducted 105 video-recorded sessions with 20 children aged 8 to 16 with PIMD/IDs. These sessions aimed to capture the emotional and behavioral states interpreted by caregivers while simultaneously collecting indoor and outdoor weather indices, location, and time data. Using cross-classified multilevel and general linear models adjusted for individual characteristics and location variability with subsequent simple slope analyses, we examined the main and seasonal interaction effects of indoor and outdoor weather indices and time of the day on the emotional and behavioral states of children with PIMD/IDs.

**Results:**

The models revealed that higher atmospheric pressure (atm), indicative of pleasant and favorable weather conditions, was associated with increased engagement (indoor: *p* < 0.01; outdoor: *p* < 0.01) and interest (outdoor: *p* < 0.01) behaviors. In contrast, engagement levels decreased before lunchtime (*p* < 0.01; *p* < 0.001), and inclement or unstable weather conditions characterized by low-pressure systems (*p* < 0.05) and stronger wind speed (*p* < 0.05) led to more refusal or disagreement. During winter, children displayed significantly more agreement with their caregivers (*p* < 0.001). Interestingly, they also engaged more on cloudy days (*p* < 0.05). Furthermore, simple slope analyses revealed that high atm conditions in fall were linked to more engagement (*p* < 0.05) while humid conditions predicted more assent behaviors (*p* < 0.001). However, cloudy weather predicted less attentional focusing (*p* < 0.05) and interest (*p* < 0.01) behaviors in winter.

**Conclusion:**

This study confirms that fluctuations in weather indices, including seasonal changes and time of the day, can provide potential pathway indicators and supplement behavioral observations to elicit the behavioral states of children with PIMD/IDs. These findings highlight the importance of considering these factors when designing meaningful interactions and communication interventions for this population.

## 1 Introduction

Children with congenital profound intellectual and multiple disabilities (PIMD) have severe or profound intellectual disabilities (IQ < 25), neuromotor dysfunctions, and comorbid chronic health, sensory, or functional impairments that severely restrict their ability to comprehend spoken or verbal language and engage in symbolic interaction with objects (1–6). Instead, they rely on subtle and refined upper and lower body movements, limbs, facial expressions, and vocalizations on a pre-or proto-symbolic level (3–5, 7–10). Consequently, eliciting their mental and emotional states due to their complex and atypical developmental trajectories and indistinct and idiosyncratic movement and behavior repertoire becomes increasingly elusive.

Understanding the communication dynamics of individuals with PIMD/IDs has greatly benefited from the introduction of Attuning Theory, as proposed by Griffiths and Smith in 2017 (11). This theory places the attuning process at its core. It describes a dynamic mechanism whereby individuals with PIMD/IDs and their caregivers continually adapt their cognitive and emotional states during communication. The theory’s framework encompasses seven interrelated categories: attuning, engagement, action, attention, stimulus, being, and setting. Within this framework, “attuning” represents the fundamental adjustment of one’s mental and emotional states in response to others, forming the cornerstone of communication dynamics. “Being,” or the emotional and mental state, impacts how each person behaves and serves as the stimulus that affects how they attend (attention), respond (action) to each other, and the nature of their interactions (engagement). Notably, “setting” emerges as a fundamental element within this framework. It refers to the complete context of a place (e.g., playground, park, or classroom) where all communication interactions transpire. It encompasses various elements like the indoor and outdoor weather conditions that fluctuate over a day during a specific season and time of the day, that significantly influences the “being,” “attention,” and “action” of individuals engaged in communication. To draw a connection, consider a scenario where a child and their caregiver are engaged in a communication interaction indoors, specifically in a classroom in the morning. The classroom’s environmental “setting” includes various elements like the availability of natural light, indoor and outdoor temperature, humidity, and atmospheric pressure. For instance, on a warm and sunny morning during fall with comfortable temperatures and moderate humidity indoors, children and their caregivers may experience a positive emotional state characterized by increased comfort and alertness. This favorable “being” state can enhance their communication, fostering positive interactions. Conversely, during inclement weather, such as a hot and humid afternoon during summer, the environmental conditions may create discomfort and irritability, which can lead to reduced and less effective communication. Incorporating weather-related indices and their fluctuations in the “setting” provides a nuanced understanding of how environmental factors, including time of the day, impact the emotional and behavioral states of individuals with PIMD/IDs and their caregivers during communication interactions.

Varying levels of physical activity and domains of children’s emotional and behavioral states related to variations in weather indices have been gaining attention for decades across settings and seasons. A repeated measures study found variations in the relationships between local weather conditions and daily accelerometer-measured physical activities (measured in counts per minute or cpm) (12). Multilevel regressions revealed that lower levels of mean cpm were associated with increased precipitation and wind speed, higher visibility (a measure of how far humans can see), and longer hours of daylight (day length) (12). In a relatively more recent study, although no distinct patterns were found in the association between season and behavior outcomes, longer day lengths were associated with lesser sedentary behavior, and age- and sex-adjusted warmer temperature and dryer weather increased moderate-to-vigorous physical activity in males (13). Moreover, in warmer seasons, such as summer and spring, higher temperature and humidity levels lead to discomfort, resulting in increased frustration, irritability, aggressive behavior, reduced prosocial behavior, and more active behavior (14–18). Conversely, during winter, elevated indoor temperature and humidity create a more comfortable environment, leading to increased alertness and reduced negative affect on others (14, 16). Increased sunshine has been associated with higher levels of prosocial behavior in children with negative affect (19). During winter, more sunshine increases enthusiasm and emotional strength and reduces frustration, sadness, and aggression (14, 15). Significant precipitation, like snow or hail, has been linked to lower levels of irritability and nervousness in children, indicating their coping mechanisms with erratic weather patterns through seeking comfort from others (14). On the other hand, higher wind speed during winter leads to decreased activity, determination, and increased fear and nervousness in children (14). Fluctuations in atmospheric pressure also influence children’s emotions, with lower pressure and higher temperature affecting activity levels, while the opposite trend predicts feelings of fear (14).

Investigating the effects of weather indices on behavior and emotional states is also pivotal in mitigating the severity of psychiatric and neurological symptoms in adults and predicting and preventing their onset and associated risks (20–27). Despite a robust body of research in the adult population, there is a notable dearth of studies examining these effects on children with neurological, motor, or physical disabilities and functional impairments. The study by VanBurskirk and Simpson, closely related to this investigation, explored whether atmospheric pressure, humidity, outdoor temperature, and moon illumination influence the behavior of three children with autism who exhibit significant challenges, including screaming, falling, head-butting, biting, kicking, hitting, and elopement (28). The limited empirical evidence to support a correlation between weather conditions or moon phases and behavior suggests that these factors may not strongly influence behavior. Nonetheless, the authors noted that methodological limitations could have affected the findings, indicating that the potential impact of weather variables on the aberrant behavior of children with severe disabilities cannot be conclusively dismissed (28). This highlights the continued interest in exploring the relationship between weather and behavior, emphasizing the need for hypothesis-driven research to justify including weather as a critical factor in assessing the emotional and behavioral states of children with PIMD/IDs.

We have developed a computer-assisted communication aid and assistive technology (AT) system that features a machine learning (ML) framework to classify and interpret the movements and behaviors of children with PIMD/IDs. Our approach leverages algorithms to understand and translate subtle physical cues into meaningful communication outputs (29). The system incorporates a comprehensive set of 53 environmental data features, including indoor and outdoor weather indices, proximity/location, and time of the day. Results from datasets recalibrated to include environmental data have shown remarkably high classification rates (over 60%). Feature selection with the Boruta algorithm indicates that approximately 70% of the environmental data features—such as time-stamp-derived data, day, GPS location data (latitude), and weather indices like UV, atmospheric pressure, temperature, humidity, and wind direction—are significant in accurately classifying movements into distinct behavioral outcome classes (29). It should be noted, however, that the Boruta algorithm’s feature selection is based on attribute weights, not index levels (high or low). Nevertheless, suppose we understand that high humidity levels, for instance, cause frustration, irritability, and aggressive behaviors in children with PIMD/IDs. In that case, the AT system can accurately detect specific emotions associated with high humidity conditions (29).

Therefore, this current study examines the main and seasonal interaction effects of indoor and outdoor weather indices, including time of the day, on the behaviors and emotions of children with PIMD/IDs. We draw upon the existing literature to formulate our hypotheses in this exploratory study. We propose that both main and seasonal interactions on the levels and fluctuations in indoor and outdoor weather indices, such as temperature, humidity, and atmospheric pressure, significantly influence the behaviors and emotional states of children. Furthermore, we posit that these effects will vary according to the time of day and may manifest seasonal patterns.

## 2 Methods

### 2.1 Participants

Twenty children (males *n* = 14; 70%) between the ages of 8 and 16 (M = 11 ± 06 years) (3rd grade to the 1st year of high school) and their direct support persons or caregivers were recruited from a special needs school. The children met the inclusion criteria: diagnosed with PIMD (*n* = 16, 80%) or severe to profound IDs (*n* = 4, 20%), some of whom had comorbid sensory impairments or chronic health conditions. The caregivers, well-acquainted and equipped with a comprehensive understanding of interpreting the children’s movements and behaviors, had supported the children for three years or more. Informed consent was obtained from the caregivers or parents, with detailed information provided about their right to withdraw from the study at any time. The study adhered to relevant international ethical guidelines and regulations, following established procedures for the study protocol, data collection, and management. This included coding and securely storing forms, data, and identifiable information on a password-protected network server database and computer to ensure confidentiality and privacy (30, 31). The study received approval from the institutional ethical board of the Faculty of Education, Ehime University, Japan (R2-18), and is part of a larger approved research project.

### 2.2 Data collection

#### 2.2.1 Session and experimental setup

During five months, from September 2019 to February 2020, we conducted 105 observation sessions, each designed to closely examine the interactions between child-caregiver dyads in natural settings. Each session followed a systematic format to capture a comprehensive view of the child’s movements and behaviors. The duration and frequency of these sessions were flexible to accommodate the specific dyad’s availability and willingness to participate, resulting in a variable number of sessions per dyad, ranging from as few as one session to as many as 15 sessions throughout the study. On average, dyads participated in approximately five sessions each. The sessions typically included a pre-observation period where caregivers were briefed about the upcoming session and their role during the interactions. These sessions usually occurred at key times, including morning greetings, lunchtime, and breaks, between 9 am and 1 pm. The interactions occurred in various locations familiar to the children, such as playrooms, classrooms, and music rooms. To accurately capture the nuances of these interactions, each session was meticulously video recorded, yielding around 30 hours of footage. The average session length was about 18.5 minutes, but this could vary significantly, ranging from as brief as approximately 0.37 minutes to as lengthy as 54 minutes, depending on the nature of the interaction.

During the sessions, various factors can influence children’s emotions and behaviors, including the interaction context, caregiver-child relationship, and current situation or task. To mitigate the impact of these variables, we employed a child-driven data collection approach. This methodology prioritizes and emphasizes interactions initiated by children rather than those initiated by caregivers or adults. In this approach, we primarily focused on capturing and analyzing instances where children take the lead in initiating communication, activities, or interactions within a given context. This means the primary data collection effort was to capture moments when the child independently expresses their emotions, desires, or intentions. Caregiver-initiated interactions, while important, were deliberately excluded or minimized during the sessions to isolate and study the child’s independent behaviors and emotional expressions without the influence of external cues or prompts from caregivers. By emphasizing child-initiated interactions, we could establish reliable associations between the child’s emotional and behavioral states and distal factors such as weather indices, seasonal variations, and other external influences.

Data collection, video recording, transcription, coding, and categorization were based on Griffiths and Smith’s theoretical and methodological framework, anchored in symbolic interactionism (11). The trained investigator, a behavior expert, targeted the typical minute and refined and subtle upper and lower body and limb movements, facial expressions, eye movements, gestures, vocalizations, and communicative interaction repertoire of children with PIMD/IDs. The behaviors were selected based on predefined lists by Griffiths and Smith (11). These provided detailed descriptions encompassing various aspects, including structural nuances such as anti and pro, negative and positive orientations. They further delved into typology, covering a broad spectrum from expressions like screaming to harmony. Additionally, these lists elaborated on indicators, comprising actions such as looking at each other, moving toward one another, smiling, maintaining close physical contact, and engaging in meaningful gazes and expressions. Furthermore, they encompassed a range of codes, including concentration, interest, and support. These codes offered insights into the manifold manifestations of each attuning category. For instance, attention was manifested through visual tracking, mobile gaze changes, fixed gaze, head positioning, and other observable cues (11). The framework of expressive behaviors by Ashida and Ishikura (32) was also utilized, which categorized expressive behaviors into six primary categories related to eye movement, facial expression, vocalization, hand movement, body posture, and noncommunicative behaviors observed in children with PIMD/IDs.

Recording was contingent upon the caregiver’s immediate interpretation of the child-initiated interaction, documented within five seconds. For instance, if a child vocalized or moved, the caregiver would respond with an interpretation, such as a verbal acknowledgment or assisting action, which was then recorded in an app called ChildSIDE (9). Each recorded entry was labeled and initially categorized according to the interaction context (e.g., calling, response, emotions, interest, negative, selecting, physiological response, positive) to easily add to the data bank or develop new ones as required. To mitigate the confounding influence of the investigator’s presence, the investigator took on a non-engaging and non-interfering role during the sessions. It is important to note that the investigator did not interact with the children before or during the sessions in a way that would disrupt or prevent the children from interacting with their caregivers. Instead, their interaction was primarily with the caregivers to understand the meaning of the movements and behaviors exhibited by the children. This collaborative approach ensured the investigator could accurately interpret the child’s actions within five seconds after initiation. This method allowed for a deeper understanding of the child’s communication attempts and behavioral expressions while minimizing any potential disruption or interference in their interactions with their caregivers. Consistency and reliability were ensured by having a single investigator conduct all sessions, thus guaranteeing the collection of accurate data from the dyads while ensuring that the investigator focused on understanding the child’s expressions and maintaining a non-intrusive presence.

**Figure 1:**
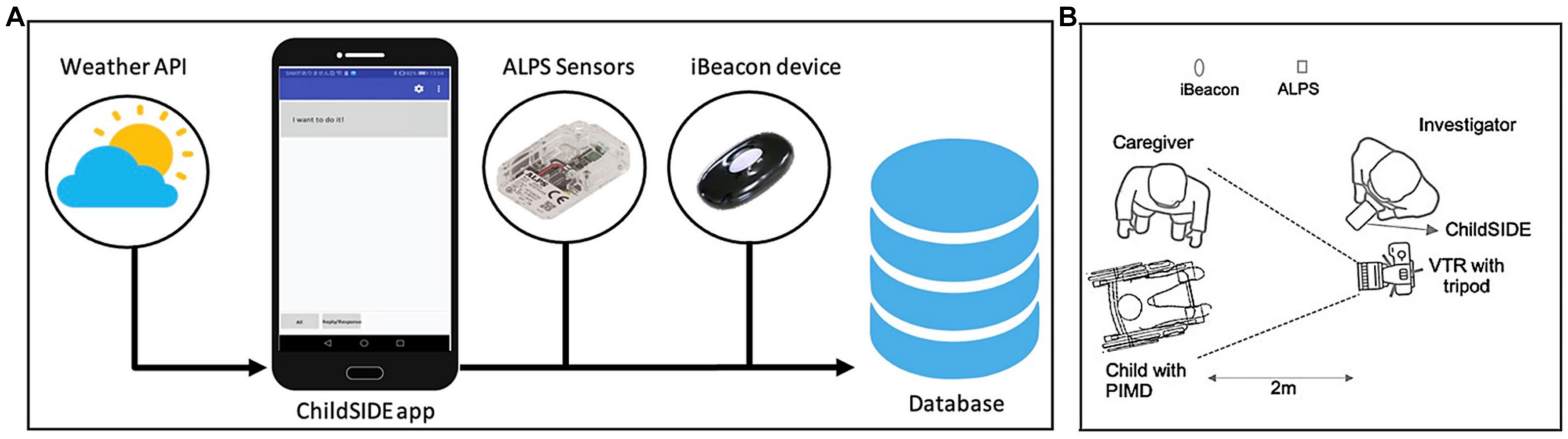
Data collection framework and experimental setup.

In addition to capturing movement and behavioral data, the ChildSIDE app seamlessly linked each data entry with corresponding environmental and locational details. The acronym “SIDE” in the app’s name stands for “Sampling Information and Data of children’s expressive behaviors and the Environment,” reflecting the app’s function of collecting comprehensive behavioral data alongside indoor and outdoor weather index values, location, time, and date stamps (as shown in Figure 1A) (9). The combined datasets were then transmitted and securely stored within a dedicated database. Indoor measurements such as UV-A intensity (mW/cm^2^), atmospheric pressure (hPa), temperature (°C), and relative humidity (RH%) were obtained in real-time using the IoT Smart Module Sensor Network Module Evaluation kit, which includes a compact multifunction Bluetooth sensor (ALPS Sensor). Outdoor weather data, like atmospheric pressure (hPa), temperature (°C), humidity (%), cloudiness (%), and wind speed (m/s), were sourced from the OpenWeatherMap API, a service that uses a reliable weather prediction model incorporating data from various sources, including global datasets (NOAA GFS 0.25 and 0.5 grid sizes, NOAA CFS, ECMWF ERA), METAR stations, weather radar data, and satellite data. Time of day and proximity location information were derived from beacons, Bluetooth low energy (BLE) devices that provide indoor positioning based on the Received Signal Strength Indicator (RSSI), ranging from –26 to –100 inches. Each of the 18 indoor locations where sessions occurred was identified by an iBeacon name.

Figure 1B shows our experimental setup in each session. The investigator utilized the app alongside a single videotape recorder (VTR) mounted on a tripod positioned at a standardized distance of two meters from the dyads. This arrangement facilitated retrospective indirect observation and inter-rater agreement analyses. To enhance data collection, one beacon and one ALPS device were strategically installed at various locations (such as shelves, blackboards, bulletin boards, or air conditioning units) approximately 2.18 meters from the app. This distance was estimated as the mean distance, with a mean error of 0.18 meters and a root mean square error (RMSE) of 0.41 meters, ensuring consistent and reliable measurements.

#### 2.2.2 Measures and preprocessing

##### 2.2.2.1 Movements, vocalizations, and behaviors

**Figure 2:**
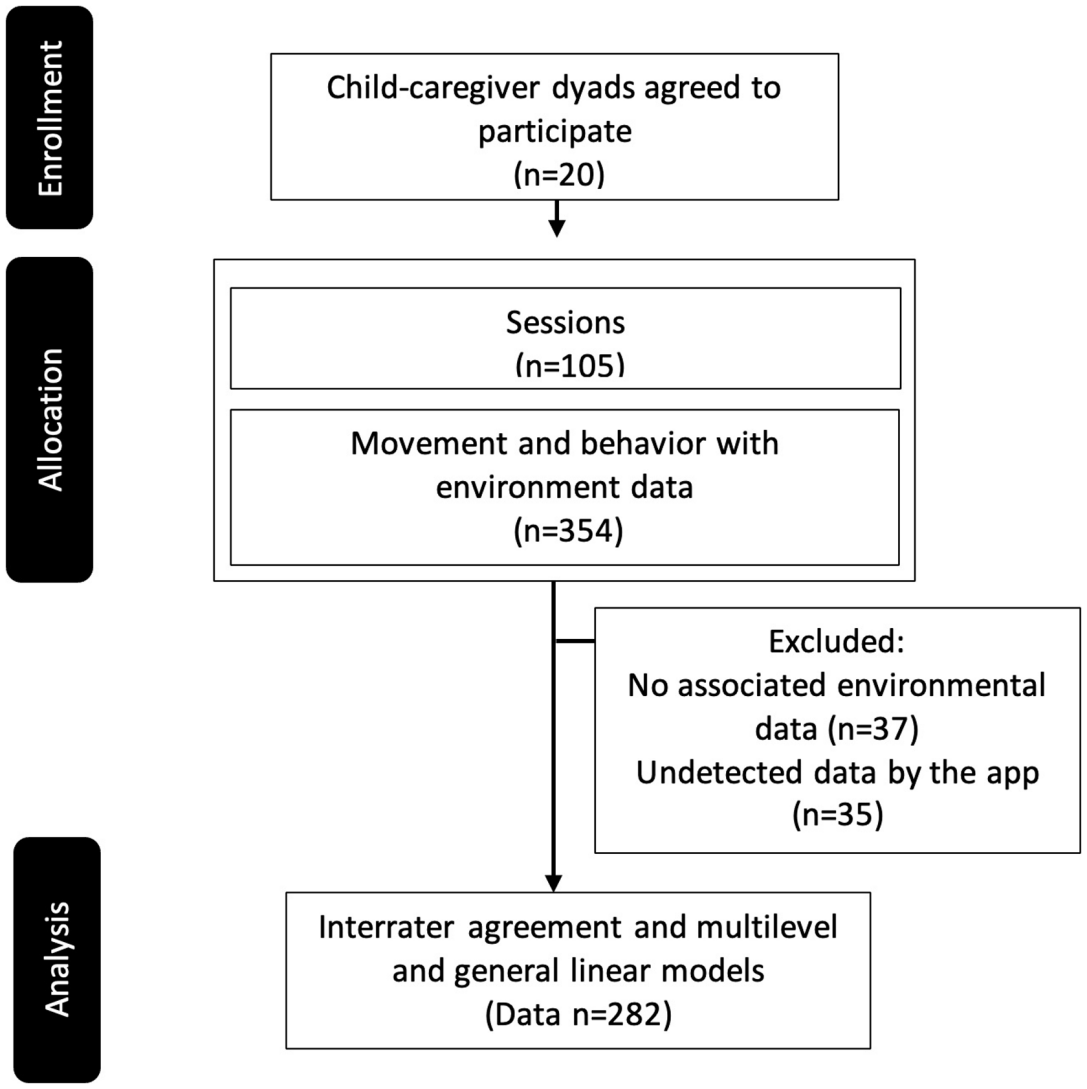
CONSORT diagram of participant, session, and data flow from enrollment, allocation, to analysis.

We collected 354 observations (average: 6, range: 1 to 34 per session) of movements, and behaviors, along with environmental data, from the app database. One behavior expert carefully reviewed the video recordings frame-by-frame at reduced speeds to ensure data validity. As illustrated in the CONSORT diagram (Figure 2), 72 data lacking behavior or associated environmental data or undetected by the app were excluded from further analysis. This resulted in 282 data points, which underwent a video-based retrospective interrater agreement analysis (*κ*). Prior to categorization, each data point was transcribed and encoded following Griffiths and Smith’s (2017) transcription and coding system, providing comprehensive and objective descriptions of the interaction context, child movements, vocalizations, behaviors, timing, and duration (based on video timestamps) (11). Verbal expressions were encoded verbatim, while non-verbal actions were sequentially described alongside the verbal ones. Vocalizations or sounds were orthographically approximated.

Subsequently, two independent behavior expert raters re-analyzed the video recordings and discussed category definitions and coding procedures. Drawing again from the structure (*anti* and *pro*, *negative* and *positive*), typology (ranging from *screaming* to *harmony*), indicators (such as *mutual gaze, physical proximity, smiles, expressions*), and codes (such as *concentration, interest, support*) of the attuning theory, the raters categorized each data point into *engagement, assent, positive, interest, refusal, disagreement*, or *disconnected/withdrawal* categories (11). Additional categories, including *attentional focusing* and *negative emotions*, were introduced as needed. Inter-rater agreement for each category was assessed using *κ* coefficients (*κ* = 0 less than chance agreement, *κ* = 0.01–0.20 slight agreement, *κ* = 0.21–0.40 fair agreement, *κ* = 0.41–0.60 moderate agreement, *κ* = 0.61–0.80 substantial agreement, and *κ* = 0.81–0.99 almost perfect agreement) with a significance level of *p* < 0.01 (33). Preliminary results are presented in Table 1, demonstrating moderate disagreements between the raters in categorizing data points related to *engagement, positive* and *negative emotions, interest*, and *disconnected/withdrawal*. The raters revisited their assessments and continued discussions until they reached a consensus, achieving almost perfect agreement levels for all categories. The final frequency distribution of each category is also presented in Table 1 (final stage).

**Table 1:** Definitions, manifestations, inter-rater agreement results, and frequency distribution of the behavior and emotional states.

Behavior and emotional states	Definition	Manifestations (sample extracts)	Initial inter-rater agreement			Final stage
			*κ*	*κ* range^a^	*p* value	*n* = 282 (%)
Engagement	The engagement of both partners in the dyad may be directed to the same focus. Verbal or non-verbal movement or behavior aims to get the attention of the caregiver or teacher to initiate an interaction and exchange words or actions (e.g., greetings, calling for attention, and response to requests).	-moves mouth to say only the “masu” part of “*Ohayo gozaimasu*” -Right hand forward, face towards the teacher. -Points a finger and whispers something. -Moves the body significantly. It takes longer for food to be chewed after it enters the mouth. -Moves the face widely. Opens mouth wide and tries to speak. Breathing becomes a little more intense. -Opens mouth wide and vocalizes repeatedly. -Waves to the camera. Turns around, laughs, and touches right shoulder with the right hand (with vocalization). Says ‘bye-bye’ while waving with both hands.	0.95	5	<0.001	50 (17.7)
Attentional focusing or concentration	In this state, the dyads are attuned and understand each other and both agreed as to what is happening and what should happen. Mostly non-verbal actions or gestures to express a decision or desire to choose between or among objects.	-Vocalizes. Cheek puffing. Eye movement. Eyebrows move. -Places hand on the card with left fingers extended. -Pulls the whiteboard closer. -Raises the corners of the mouth. Selects a card with the right hand. -Pulls the whiteboard closer. Moves gaze (in line with the card). -Moves gaze in line with the colored pencils. (Face together). Grabs the colored pencils. -Shakes head sideways. Gazes between desk and chair.	1.00	5	<0.001	35 (12.4)
Assent	Demonstrates attuned agreement between the dyad. The child carries out an action or asks a question or puts a demand on the caregiver. Then the caregiver confirms the child’s need or want by asking question then the child attunes (agree or approve) and responds.	-Mouthing away from the cup. Eye movement. Moving the head upwards. -Vocalization. Eye movement. Moves face down to the right. Extends left arm. -Points to somewhere in the picture book with the left hand and pushes. -Vocalizes ‘yes’. -Looks at the teacher’s face and vocalizes ‘yes’. Makes a slight nodding motion.	1.00	5	<0.001	68 (24.1)
Positive emotions	Mostly non-verbal expressions of feelings of being happy or pleasant (“delicious,” “I like…”), excited, or enthusiastic (“fun”).	-Turns towards the sound and directs attention to the book. When doing so, opens mouth wide. Appears to be smiling. -Smiles. Smiles at the potatoes. -Raises the corners of mouth and shakes face from side to side while holding the back of head with the right hand. -Occasionally points at the camera and raises the corners of his mouth. Shakes face from side to side. -Opens mouth and brings hand to the mouth. Looks at the teacher and opens eyes. Raises eyebrows upwards. -Reaches right hand behind back. Smiles. Rocks body back and forth. Squeaks feet.	0.91	5	<0.001	23 (8.16)
Negative emotions	Mostly non-verbal movements and behaviors that demonstrate feelings of being anxious or worried, anger, or pain.	-Looks away from the teacher. -Shake the head vertically. Eye movement. Turns the head downwards. -Shakes the body. -Face is straining. -Body begins to sway. Corner of mouth and eyebrows droop. Suddenly stands up and walks towards the TV. -Frowns, touches the teacher’s hand with the right hand.	0.74	4	<0.001	10 (3.55)
Interest	Demonstrates an obvious attention in (attuning to) an object (“show me”), action, or doing an action (“I want to do!”).	-Vocalizes. Eye movement. Movement of face to the right. -Eye movement (according to the card). Movement of hands. -Face movement (to match the card). Looks to the right of the screen. Moves body closer to the chair. Tilts head back, extends left arm, and says ‘quickly’. Vocalizes. Lowers mask and licks right hand. Looks at the teacher. Calls out ‘good’ repeatedly. -Gazes at the tablet. Changes posture to look at it. Looks at the screen.	0.71	4	<0.001	61 (21.6)
Refusal or disagreement	The dyad does not accede to the wishes of the other so the interplay between the dyad is negative. Verbal or non-verbal actions and vocalizations (“no,” or “no, it’s different”) to express refusal or disagreement.	-Closes mouth when bean curd is brought close. Sticks out tongue. Turns face away. -Produces loud voice to interrupt teachers. Moves hands loudly. -Touches face (mouth and nose) with hand while moving fingers. -Tries to brush away the teacher’s hand. Moves the mouth. Vocalizes “no.” Tilts upper body to the left. -The teacher asks, “This?” In response, looks at the castanets and then looks down. Reaches for the castanet he wants.	1.00	5	<0.001	19 (6.74)
Disconnected or withdrawal	A low or absent level of attuning, combined with little or no cooperation or greater degree of separation is occurring between the partners which may be due to internal (e.g., sleepy, tired, or hungry) or external (e.g., cold) stimulus.	-Closes eyelids. Looks up and does not move. -Eyes closed and looking up. -Tiny mouth opening. Does not accept food in mouth. -Tries to look up and close eyes. Blinks for a long time. -Looks downwards. Movement stiffens. -Touches the left hand in front of the nose and the right hand towards the mouth. Left fingers move slightly. Eyes appear to be barely open.	0.83	5	<0.001	16 (5.67)

a Inter-rater agreement for each category was assessed using *k* coefficients (*k* = 0 less than chance agreement, *k* = 0.01-0.20 slight agreement, *k* = 0.21-0.40 fair agreement, *k* = 0.41-0.60 moderate agreement, *k* = 0.61-0.80 substantial agreement, and *k* = 0.81-0.99 almost perfect agreement) with a significance level of *p* < 0.01 (33).

Table 1 also shows the behavior and emotional states and their definitions and manifestations. The *engagement* category in this context pertains to the active interaction between children and their caregivers. It involves the child’s efforts to capture the caregiver’s attention and initiate communication or interaction. These efforts encompass a variety of behaviors, both verbal and non-verbal. For instance, the child may use verbal engagement by making specific sounds or vocalizations, even if limited, to signal their desire for interaction. Additionally, physical movements and gestures play a significant role. The child might turn their body toward the caregiver, point at something, or wave to establish a connection. The *attentional focusing* or concentration category pertains to attunement and mutual understanding between the children and their caregivers. During this state, both parties share an understanding of the ongoing situation and mutually agree on what actions or decisions should be taken. The focus in this category primarily involves non-verbal actions and gestures used to express a decision or desire, often related to choices among various objects or options. Some observable behaviors associated with this state of attentional focusing or concentration include vocalizations, such as making sounds or vocal cues, and physical expressions like cheek puffing, eye movements, and eyebrow movements, which can indicate the child’s engagement and decision-making process. The *assent* category represents a state of attuned agreement within the dyad. In this dynamic, the child initiates an interaction by performing an action, asking a question, or making a demand. The caregiver then acknowledges the child’s communication by asking a clarifying question, to which the child responds affirmatively or with approval, indicating attunement and agreement. For instance, the child may turn their head upwards and mouth away from a cup, a gesture often accompanied by eye movement, possibly indicating a preference or refusal without verbalization. In another interaction, the child might vocalize while simultaneously shifting their gaze downward to the right and extending their left arm, suggesting a specific need or request is being communicated. Furthermore, it is essential to comprehend and discern the children’s emotional states within the context of the dyadic interactions. Two significant categories encapsulating these emotional states are positive and negative emotions. *Positive emotions* encompass an array of non-verbal expressions denoting happiness, delight, excitement, or enthusiasm. These emotions manifest through observable actions, such as turning toward sources of interest, genuine smiles, playful facial movements, and expressions of joy. Conversely, *negative emotions* are characterized by non-verbal movements and behaviors that convey feelings of anxiety, worry, anger, or pain. Children in this emotional state may exhibit signs such as avoiding eye contact, shaking their heads, displaying physical tension, or showing facial expressions of distress. Additionally, they may engage in sudden movements or seek physical contact for comfort.

Clear signs of attention and engagement with particular objects, actions, or activities define the *interest* category. Children in a state of interest typically express their engagement through vocalizations and eye movements, which are directed toward the object or activity that captures their attention. For instance, when a child demonstrates interest, they may vocalize their excitement or desire, accompanied by eye movements that indicate their attention. Additionally, they may adjust their body position or gaze to align with the object or action of interest. In contrast, signifying moments when there is an evident lack of agreement or compliance within the dyad, *refusal, or disagreement* interactions are characterized by verbal or non-verbal actions and vocalizations that express a refusal or disagreement with the actions, suggestions, or requests of the other person in the dyad. When a child displays refusal or disagreement, they may use a variety of communication cues to convey their dissent. These cues include closing their mouth, sticking out their tongue, turning their face away, or producing vocalizations like “no” to express their refusal. Physical actions such as attempting to brush away a caregiver’s hand or moving their body in a different direction may further communicate their disagreement. Lastly, *disconnected* *or withdrawal* is a category that describes the state in which there is a low or absent level of attuning between the partners in a dyad, resulting in minimal cooperation or a significant degree of separation. This *disconnection or withdrawal* can be influenced by various factors, including internal states (such as feeling sleepy, tired, or hungry) or external stimuli (such as feeling cold). For instance, in this category, observable behaviors and movements typically indicate a lack of engagement or interaction. These behaviors may include closing eyelids, looking upward without movement, keeping the eyes closed, making minimal mouth movements, or exhibiting stiffness in movement. These signs suggest that the child is disengaged or withdrawn from the ongoing interaction with their caregiver or partner in the dyad.

##### 2.2.2.2 Environment measures

The special needs school from which the dyads were recruited is located near the border of Toon and Matsuyama cities, situated atop the alluvial fan of the Dogo Plain in the Ehime prefecture on the island of Shikoku, in the southwest of Japan. These cities have a humid subtropical climate (Cfa according to the Köppen-Geiger classification), characterized by mild to warm temperate summers and cool winters with occasional light snowfall. Annual temperatures typically range from a low of 2.8°C to a high of 25°C, averaging 13.9°C. In January, the average low is 5.3°C; in August, the average high reaches 27°C. Humidity levels usually vary from 72% in the spring to 84% in the summer. The average yearly precipitation is 112.6 millimeters (4.43 inches), with summer experiencing the highest rainfall, sometimes up to 10 inches. Monthly sunshine hours span from 7.2 to 13.7. The windiest months are from October to April, with average wind speeds often exceeding 7.0 miles per hour.

To include all movement and behavior data with a complete set of environmental data (complete cases) and to minimize errors in multilevel models, missing data points for single or multiple locations (beacon; *n* = 45), indoor UV data (*n* = 98), and outdoor atmospheric pressure, temperature, humidity, cloudiness, and wind speed (*n* = 14) were imputed. The missing values were replaced either with the nearest (proximity) values or with an estimated value calculated from the mean of the available or non-missing data points using the *k*-Nearest Neighbor (*k*-NN) imputation method (*k* = 14) (34).

### 2.3 Statistical analysis

The effects of indoor and outdoor weather indices on children’s emotional and behavioral states were analyzed, accounting for individual characteristics and location variability. A cross-classified non-hierarchical multilevel model was used, employing the R function *glmer* from the *lmerTest* package. This approach expands upon hierarchical multilevel modeling, typically used in studies where data have a nested structure (for example, measurement occasions nested within children, who are nested within teachers). Cross-classification allows for the analysis of lower-level units associated with combinations of higher-level units formed by crossing multiple classifications (16, 35). In this study, each observation session (the level 1 unit) was associated with a specific child (identified by an ID number), who may vary in age, gender, educational level, and condition, and observed in one of the 18 designated indoor locations. Consequently, the data are cross-classified, with intersecting sessions, locations, and child characteristics. The session is the level 1 unit, while factors such as age (in years), gender (male or female), condition (severe/profound IDs or PIMD), education level (elementary, junior high, or senior high school), and indoor locations (classrooms, music rooms, and playrooms) are included as level 2 measures (random effects).

In the analysis, the null model was a random intercept-only model, which accounted for the variability in children’s characteristics and location data. Fixed effects included hourly indoor weather indices (UV intensity, atmospheric pressure, temperature, and humidity) and outdoor weather indices (atmospheric pressure, temperature, humidity, cloudiness, and wind speed), as well as for seasons (fall and winter, with fall as the reference season) and time of day (hourly from 9 am to 1 pm, with 9 to 10 am as the reference period). To mitigate multicollinearity due to the high correlation between indoor and outdoor atmospheric pressure and temperature, separate models were fitted for indoor and outdoor predictors. Interaction terms between predictor variables and season were also included to explore the relationship between changes in weather indices across seasons and variations in the emotional and behavioral states of the children.

Before entering predictor variables into the models, continuous variables (indoor UV, indoor and outdoor atmospheric pressure, temperature, and humidity, and outdoor cloudiness and wind speed) and dummy-coded categorical variables (seasons and time intervals) were centered to mitigate multicollinearity and facilitate *post hoc* analyses of seasonal interaction effects (36). Model comparisons were conducted with a null or baseline model to estimate the degree of dependence of the variance in a *Poisson* distribution, representing the difference between the lower and upper limits in a 95.4% range that includes the expected values (intraclass correlation coefficient or ICC). If the difference between the upper and lower limits exceeded one at level 2 (children or location), multilevel linear models were applied; otherwise, general linear models were used for differences less than one. All predictor variables were subsequently included in the model, followed by the Variance Inflation Factor (VIF) estimation to address the impact of multicollinearity. Predictors with VIF values greater than five were excluded from the model. *p*-values of all predictor variables were adjusted for *post hoc* alpha-level correction using the false discovery rate (FDR) or the Benjamini-Hochberg method. When significant seasonal interactions were found (*p* < 0.05) (FDR 0.5), simple slope analyses were carried out to explore the nature of these interactions.

## 3 Results

### 3.1 Baseline indoor and outdoor weather data

**Figure 3:**
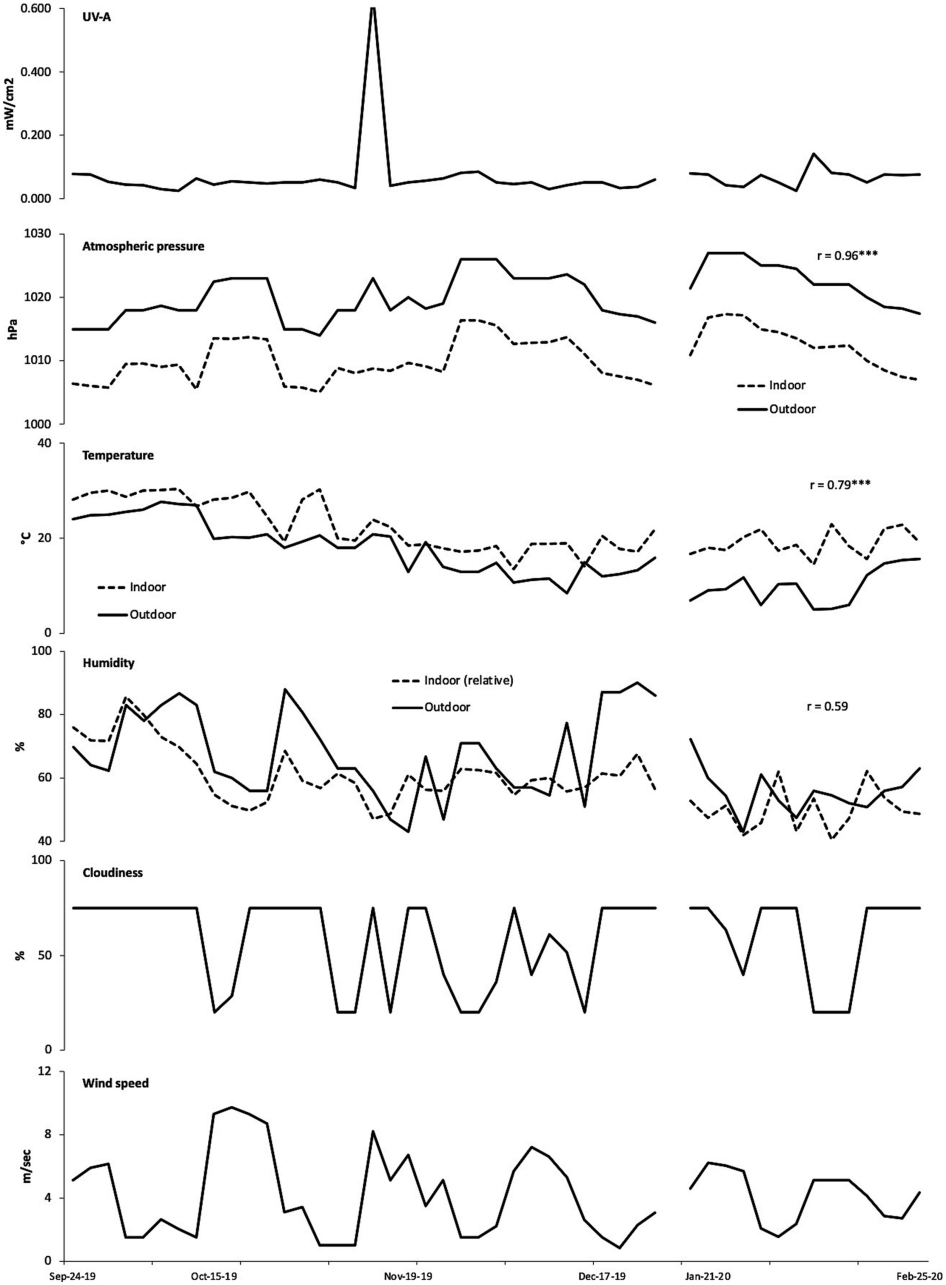
Hourly mean indoor and outdoor weather indices across the study period. *p < 0.05, **p < 0.01, and ***p < 0.001.

Figure 3 presents the hourly real-time mean values of indoor and outdoor weather indices and movement and behavior data collected throughout the study, covering both fall (September 24 to December 17, 2019) and winter (January 14 to February 25, 2020) seasons. The average indoor UV-A index values (0.07 mW/cm^2^) remained consistent throughout both seasons, ranging from 0 to 20.48 mW/cm^2^. However, a notable outlier occurred on November 12 at 11:22 am, when the UV-A index abruptly increased, reaching a maximum value of 0.64 mW/cm^2^. Although this peak value stood out, it was retained in the analysis since it did not appear to be an erroneous measurement. Concurrently, there were also sudden fluctuations in indoor temperature (19 to 23.9°C) and humidity (58.5 to 47%), as well as outdoor temperature (18°C to 20.8°C), humidity (63 to 56%), cloudiness (20 to 75%), and wind speed (1 to 8.20 m/sec). While there was no definitive explanation for these fluctuations, they were considered valid data points.

Atmospheric pressure, measured in hectopascals (hPa), indicates weather patterns, where high-pressure systems are typically linked with clearer skies and stable weather, and low-pressure systems often bring cloudiness, wind, and precipitation. In our study, there was a strong correlation between indoor and outdoor atmospheric pressure (*r* = 0.96, *p* < 0.001). The slight average pressure differences between indoor (higher by approximately ten hPa) and outdoor environments and across seasons (with a three hPa difference between fall and winter) suggest that external air could readily enter through windows, influencing indoor ventilation, air quality, and potentially affecting weather sensitivity symptoms (37). Indoor temperature levels maintained a higher average (19 to 22°C) compared to the outdoors (9.8 to 16°C) during both seasons, with a notable correlation between indoor and outdoor temperatures (*r* = 0.79, *p* < 0.001). The relative humidity (RH%), which measures water vapor in the air against the temperature, showed an inverse relationship with outdoor humidity levels (60 to 69%), generally surpassing indoor levels (50 to 62%). Higher humidity was noted in fall (average: 62 to 68%) compared to winter (average: 50 to 60%). Cloudiness, the proportion of the sky obscured by clouds, did not show significant variations between fall and winter, averaging 42 to 51%. Wind speed, the velocity of air movement, also remained consistent across both seasons, with average speeds ranging from 1.82 to 2.03 m/sec.

### 3.2 Main and seasonal interaction effects

Tables 2 and 3 detail two-level models that examine the main effects and seasonal interactions of indoor and outdoor weather indices on children’s behavioral and emotional states. Variance analysis, considering the children’s age, gender, education level, and conditions, indicates that these factors do not significantly affect the behavioral and emotional states under investigation. For categories such as attentional focusing, positive emotions, interest, and disconnected states, the variance between location units is greater than one, suggesting the suitability of two-level models to estimate coefficients and standard errors accurately. In contrast, general linear models are deemed appropriate for analysis of engagement, assent, negative emotions, and refusal, where the differences are less than one.

**Table 2:** Multilevel liner model coefficients and standard errors of the main and seasonal interaction effects analysis of indoor weather indices and time of the day on the behavior and emotional states.

Predictor variables	Engagement^a^		Attentional focusing		Assent^a^		Positive emotions		Negative emotions^a^		Interest		Refusal^a^		Disconnected	
	C	SE	C	SE	C	SE	C	SE	C	SE	C	SE	C	SE	C	SE
Time (10 to 11 am)	−0.73	0.38	1.13	0.79	0.74	0.46	–	–	−0.15	0.87	–	–	−1.20	1.30	−0.94	1.24
11 to 12 pm	−2.03	0.64**	0.46	0.85	0.57	0.48	–	–	0.11	0.87	–	–	−0.22	1.13	−0.68	1.31
12 to 1 pm	0.18	0.44	1.17	0.83	0.97	0.51	–	–	−18.00	2497.1	–	–	1.81	1.04	2.78	1.03
Season (winter)	0.54	0.52	0.82	0.83	1.22	0.51	−2.08	1.19	−2.09	1.21	−0.21	0.58	1.13	1.06	−0.02	1.34
UV (mW/cm2)	0.14	0.26	0.92	0.43	−0.40	0.85	−1.43	1.21	0.04	0.22	−1.53	0.97	0.05	1.24	0.58	0.41
Pressure (hPa)	0.64	0.20**	0.13	0.30	−0.16	0.18	−0.41	0.34	−0.27	0.42	0.56	0.21	−0.90	0.51	−0.13	0.46
Temperature (°C)	0.14	0.18	0.29	0.31	0.02	0.16	−0.52	0.61	−0.50	0.42	0.52	0.33	0.07	0.37	−0.90	0.38
Humidity (%)	0.12	0.22	0.45	0.34	−0.29	0.22	−1.14	0.63	−0.61	0.64	−0.10	0.22	0.64	0.39	1.42	0.60
Season*UV	–	–	3.21	1.31	2.54	1.36	−2.12	2.78	–	–	−0.85	2.06	1.26	2.56	–	–
Season*pressure	−0.78	0.36	−0.22	0.53	−0.78	0.30	−1.71	0.82	–	–	−0.09	0.41	−0.22	0.95	−0.27	1.00
Season*temperature	–	–	–	–	–	–	−2.69	1.55	–	–	1.07	0.85	–	–	–	–
Season*humidity	0.16	0.51	0.94	0.99	0.70	0.45	–	–	–	–	–	–	–	–	−0.58	1.34

C, coefficient; SE, standard error; −, not included in the final model due to high (≥5) VIF.

a The expected value of the frequency of the outcome differs by <1 between the upper and lower limit in level 2, thus coefficients and standard errors were computed in general linear models; Reference levels for the categorical variables = Time: 9 to 10 am; Season: Fall; *p*-values: *p < 0.05, **p < 0.01, and ***p < 0.001 were false discovery rate (FDR) adjusted.

**Table 3:** Multilevel liner model coefficients and standard errors of the main and seasonal interaction effects analysis of outdoor weather indices and time of the day on the behavior and emotional states.

Predictors	Engagement^a^		Attentional focusing		Assent^a^		Positive emotions		Negative emotions^a^		Interest		Refusal^a^		Disconnected	
	C	SE	C	SE	C	SE	C	SE	C	SE	C	SE	C	SE	C	SE
Time (10 to 11 am)	−0.83	0.39	0.11	0.74	0.89	0.49	1.42	1.19	−0.10	0.95	−0.01	0.42	−1.58	1.31	−1.02	1.27
11 to 12 pm	−2.60	0.65***	−0.91	0.79	1.05	0.55	1.21	1.21	−0.03	0.89	−0.76	0.47	−0.89	1.14	−0.51	1.43
12 to 1 pm	−0.50	0.44	−0.35	0.79	0.96	0.51	2.82	1.21	−17.5	2483.9	0.13	0.47	1.11	1.02	1.56	1.21
Season (winter)	−0.02	0.52	0.52	0.72	2.31	0.51***	−0.11	0.88	−2.06	1.24	0.08	0.54	0.78	0.94	−0.97	1.68
Atmospheric pressure (hPa)	0.64	0.24*	−0.27	0.30	−0.31	0.22	0.09	0.40	0.12	0.62	0.75	0.22**	−1.74	0.59*	−0.62	0.65
Temperature (°C)	0.20	0.26	0.53	0.39	0.09	0.27	0.03	0.58	−0.48	0.47	0.49	0.22	−0.28	0.57	−0.64	0.86
Humidity (%)	−0.33	0.24	−0.69	0.30	0.35	0.27	0.43	0.45	0.00	0.52	−0.03	0.25	0.30	0.53	−0.20	0.75
Cloudiness (%)	0.42	0.17*	0.14	0.31	−0.45	0.20	0.26	0.46	0.97	0.63	0.39	0.20	−0.14	0.46	−0.10	0.42
Wind speed (m/s)	0.18	0.19	–	–	−0.12	0.17	0.42	0.41	0.23	0.54	−0.26	0.22	1.44	0.51*	−0.06	0.62
Season*Atmospheric pressure	−1.00	0.38*	−0.74	0.73	−0.39	0.34	–	–	–	–	−0.19	0.44	–	–	−0.53	1.38
Season*temperature	–	–	–	–	–	–	–	–	–	–	–	–	0.18	1.07	–	–
Season*humidity	−0.24	0.59	–	–	2.41	0.55***	–	–	–	–	–	–	−1.07	1.55	0.31	2.23
Season*cloudiness	–	–	−1.70	0.58*	–	–	–	–	–	–	−1.52	0.43**	–	–	–	–
Season*wind speed	–	–	–	–	−0.57	0.59	–	–	–	–	−1.31	0.62	–	–	–	–

C, coefficient; SE, standard error; −, not included in the final model due to high (≥5) VIF.

a The expected value of the frequency of the outcome differs by <1 between the upper and lower limit in level 2, thus coefficients and standard errors were computed in general linear models; Reference levels for the categorical variables = Time: 9 to 10 am; Season: Fall; *p*-values: *p < 0.05, **p < 0.01, and ***p < 0.001 were false discovery rate (FDR) adjusted.

#### 3.2.1 Indoor weather indices

After excluding predictor variables with VIF values exceeding five and performing alpha-level correction using the FDR, it is observed that indoor weather indices did not show significant main or seasonal interaction effects on the behavior and emotional states. The notable exception was the impact of atmospheric pressure on engagement behaviors, as presented in Table 2. A higher indoor atmospheric pressure positively influenced engagement, implying that children interacted more with their caregivers when the indoor pressure was elevated. Specifically, a 10% increase in indoor pressure correlated with a 0.64-point increase in engagement behaviors (*p* < 0.01). Additionally, a significant decrease in engagement was observed during the 11-12 pm time interval (-2.03, *p* < 0.01), suggesting that children were less engaged during this hour compared to the baseline reference time of 9–10 am.

#### 3.2.2 Outdoor weather indices

As reported in Table 3, outdoor indices show trends similar to those observed with indoor indices, particularly regarding atmospheric pressure. Higher levels of outdoor atmospheric pressure were positively correlated with increased initiation and engagement behaviors among children and their caregivers (0.64, *p* < 0.01). Similar evidence suggests that cloudiness predicts higher levels of engagement, with a greater percentage of cloudiness or cloud cover being associated with increased engagement by 0.42 points *p* < 0.05). On the other hand, the time variable exhibited negative effects on engagement behaviors. Specifically, compared to the reference time interval of 9 am to 10 am, children demonstrated lower levels of engagement with their caregivers from 11 am to 12 pm (-2.60, *p* < 0.001). A statistically significant and robust effect of season on assent behaviors was also observed, with children showing higher levels of assent during winter (2.31, *p* < 0.001). Furthermore, outdoor atmospheric pressure was predictive of interest and refusal behaviors. Increasing outdoor atmospheric pressure was associated with a significant increase in interest behaviors (0.75, *p* < 0.01), while also corresponding to a decrease in refusal or disagreement behaviors (−1.74, *p* < 0.05). Wind speed also significantly impacted refusal behaviors; a 10% increase in wind speed led to a 1.44-point increase in negative responses or refusal behaviors towards caregivers (*p* < 0.05).

### 3.3 Simple slope results on seasonal interaction effects

**Figure 4:**
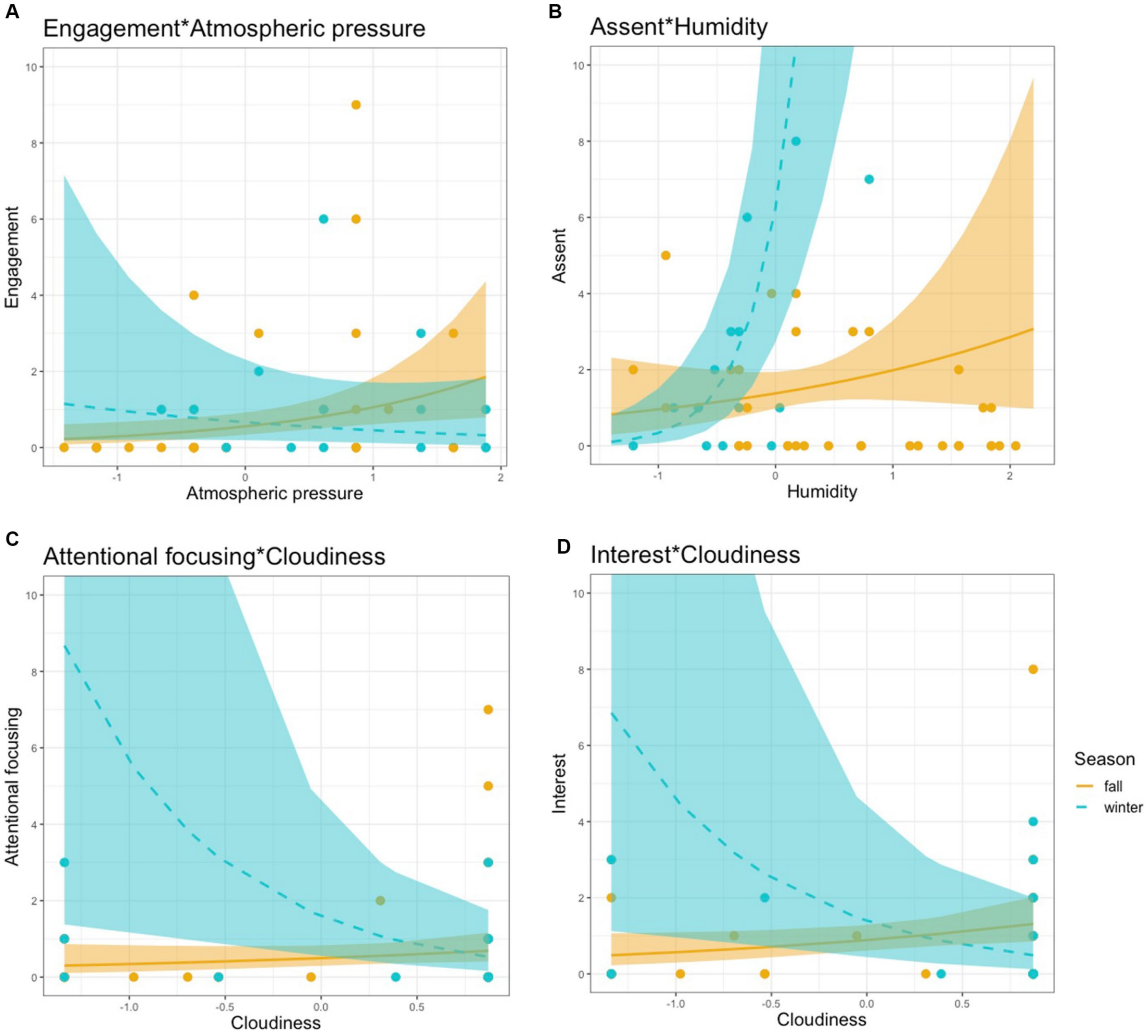
Simple slope analyses results on the seasonal interaction effect of outdoor weather indices on the behavior and emotional state outcomes.

Upon detecting significant seasonal interactions (*p* < 0.05), as indicated in Table 3, simple slope analyses were performed to explore the nature of these interactions. Figure 4 illustrates the results of these analyses, depicting the effects of outdoor weather indices on behavioral and emotional outcomes during fall and winter. Specifically, an increase in outdoor atmospheric pressure during fall was associated with a marked increase in engagement, with children initiating more interactions with their caregivers (0.64, SE: 0.24, *p* < 0.01), as shown in Figure 4A. Humidity had a significant influence on assent behaviors, with a robust interaction effect of 2.77 (SE: 0.68, *p* < 0.001) during winter, indicating that children were more likely to show agreement with their caregivers in more humid conditions during the cold season, as illustrated in Figure 4B. Conversely, cloudiness was shown to have a negative impact on attentional focusing (-1.55, SE: 0.61, *p* < 0.05) and interest (−1.13, SE: 0.48, *p* < 0.05), as shown in Figures 4C and 4D, respectively. This suggests that increased cloudiness in winter may lead to decreased attentiveness and interest among the children.

## 4 Discussion

In our study, we sought to explore the role of environmental factors, specifically indoor and outdoor weather indices, as potential pathway indicators to the occurrence or changes in emotional and behavioral states of children with PIMD/IDs. We employed multilevel and general linear model analyses to adjust for individual characteristics and variations in location. Our findings suggest that the occurrence or variations in the behaviors and emotions were strongly associated with levels and fluctuations in outdoor weather indices. Notably, the children’s responses, reflected in behaviors such as engagement, attentional focusing, assent, interest, and refusal, were markedly responsive to variations in specific weather indices such as atmospheric pressure, cloudiness, wind speed, and humidity. Moreover, our analysis revealed that seasonal changes in these weather indices and the time of day played a significant role in molding the behavioral responses of children with PIMD/IDs.

Among the indoor and outdoor weather variables, atmospheric pressure emerges as a significant indicator of children’s behavioral responses. Children demonstrated heightened attentiveness and interest in periods characterized by high atmospheric pressure, which often coincide with pleasant and sunny weather conditions. Likely, the observed behavioral changes were not directly caused by pressure but rather by the overall environmental conditions accompanying it. It is plausible that when these children experienced pleasant and sunny weather conditions, it caused them to have a more relaxed and comfortable state, though challenging to express verbally, it might have been reflected in more positive behaviors such as heightened interest and improved attentional focus. Moreover, during sunny weather, children may also have more opportunities for interesting activities, making them more attentive to their interactions and surroundings. This heightened engagement with the environment and increased social interaction during favorable weather could account for the observed behavioral improvements. The results also revealed that seasonal variations play a role, particularly during fall when high atmospheric pressure levels were associated with increased engagement behaviors. For instance, the sunny and cooler days of fall might create a more comfortable and inviting environment for engaging activities and interactions with caregivers. Consequently, children might be more inclined to engage with their surroundings and exhibit more positive behaviors during this season. Additionally, it is possible that caregivers’ responses to pleasant weather conditions also influence these behavioral occurrences. Caregivers might be more inclined to organize indoor and outdoor activities during such weather, providing more opportunities for interaction and engagement.

Adverse weather conditions characterized by low-pressure index levels and strong winds significantly contributed to increased refusal or disagreement behaviors among the children. Such weather often brings about less pleasant conditions, like colder temperatures or precipitation, which can confine activities indoors, potentially leading to frustration or restlessness among the children. Due to their limited verbal communication abilities, children with PIMD/IDs might express sensory discomfort or displeasure through non-verbal behaviors, such as refusal or disagreement, to communicate distress. Furthermore, these behaviors were also observed during periods with moderate to strong winds, which brought colder weather within the study period and created less favorable conditions. Wind can cause unusual or uncomfortable sensations on the skin and discomfort in the ears or sinuses, which may be particularly distressing for children with sensory sensitivities. This physical discomfort could lead to increased refusal or disagreement behaviors as children try to express their discomfort or pain, resulting in less interaction with their caregivers. Prior studies have similarly linked rising wind speeds to decreased physical activity, passivity, lack of determination, fear and nervousness during colder and less favorable weather conditions among children, and depressive symptoms and panic episodes among adults (14, 38–45).

The impact of high cloud cover on engagement behaviors among children revealed some intriguing and initially counterintuitive findings. Surprisingly, children demonstrated increased engagement as cloud cover levels rose. This finding appears contradictory, considering that high cloud cover typically signifies a low-pressure system, and adverse weather conditions are often seen as less favorable for activities and interactions. Despite this, the analysis also showed increased atmospheric pressure, typically related to fairer weather conditions. This suggests there might have been fluctuations in weather conditions, with intermittent cloud cover and periods of sunshine. The intermittent sunlight penetrating through the clouds could have potentially had a stimulating effect on the children’s mood and energy levels, thus encouraging more engagement. It is essential to consider that sunlight contributes to the total hours of sunshine, and previous research has indicated that increased sunshine hours are linked with elevated enthusiasm and vigor in children (14). However, although indoor UV radiation did not emerge as a significant predictor of behavioral outcomes, we speculate that the shorter sunshine hours in winter and reduced exposure to natural light sources on cloudy days decrease the attentional focusing and interest behaviors among the children with PIMD/IDs. The additional impact of extended cloud cover during winter, which leads to a colder environment, likely exacerbates this effect, resulting in decreased task-oriented behavior (16). Previous investigations have found that when visibility and daylight hours were improved, children exhibited higher physical activity levels and reduced sedentary behavior, particularly during the late afternoon and early evening or longer day lengths (12, 13, 46, 47). Regarding social behaviors, children prone to higher levels of negative affect displayed increased prosocial behaviors in the presence of greater sunshine (19). Moreover, compared to students in non-daylit classrooms, children exposed to daylight exhibited enhanced social behaviors and cognitive skills, indicating higher overall development (48). These findings underscore the importance of natural light exposure, especially during the winter months, in positively influencing the behavior and well-being of children with PIMD/IDs.

Another interesting finding highlighting the importance of considering the time of day in studying the behavioral states among children with PIMD/IDs is the apparent decrease in engagement behaviors, showing significantly less arousal and less inclined to engage with their caregivers from 11 am to 12 pm. This could be attributed to various factors, such as hydration levels or eating patterns, as suggested by previous research. During this hour, children may already be motivated to eat or drink, and they may also have opportunities for physical activity or play, diverting their attention away from engaging with caregivers. Furthermore, it is worth noting the significance of recess or breaks in promoting attentiveness and productivity in the classroom and social-emotional learning and growth among children. Unstructured breaks or recess periods, particularly those observed in the special needs school where the children with PIMD/IDs were recruited, provide valuable opportunities for social interactions and the practice of movement and motor skills. These interruptions during unstructured time help children reduce stress and distractions, ultimately enhancing their cognitive abilities (49). While this concept requires further validation, specifically among children with PIMD/IDs, it is worth considering that caregivers or teachers could incorporate structured physical activities or games before lunchtime to stimulate the children’s engagement and foster a more productive and interactive environment.

Existing studies previously identified outdoor humidity as a reliable predictor of children’s emotional states during winter. Ciucci and colleagues found that higher outdoor humidity on winter days was associated with increased frustration, sadness, and aggression when children’s ongoing tasks were interrupted or their goals were blocked (15, 16). Similarly, Lagacé-Séguin and Coplan observed that fluctuations in humidity levels significantly predicted negative emotions, including irritability and hostility, among children during winter (14). Contrary to these assumptions, our findings did not support the impact of outdoor humidity on children’s emotional states. Instead, we found a robust positive effect of increasing outdoor humidity during winter on the assent behaviors of children with PIMD/IDs. This suggests that, in the context of our study, a comfortably humid environment during winter may have a soothing or comforting effect on these children, potentially mitigating any negative emotional responses they might experience in other circumstances. This finding may appear counterintuitive since one might expect indoor humidity to influence the assent behavior outcomes, given that data were collected indoors. However, our analysis revealed no correlation between indoor and outdoor humidity indices in contrast to previously observed correlation even at cooler temperatures (50). Therefore, we cannot directly infer how outdoor humidity directly affected the children in our study. The absence of a direct correlation between indoor and outdoor humidity indices in our analysis highlights the complexity of indoor environmental conditions. Even though data were collected indoors, indoor humidity levels may not have been directly influenced by outdoor conditions. Indoor environments are subject to various factors, including heating, ventilation, and air conditioning systems, which can affect humidity independently of outdoor conditions. Thus, this finding can be interpreted in light of the lower threshold of outdoor humidity (43%), which falls within the range of acceptable or comfortably humid environments (30 to 60% relative humidity). It is possible that within this range, variations in outdoor humidity had a positive impact on the children’s behavior, promoting a sense of well-being and agreement or assent behaviors.

Several studies have delved into understanding the behaviors and communication attempts of children with PIMD, particularly emphasizing the interplay between physiological indicators of autonomic activity, physical movement, and behavioral indicators (51–53). For instance, in a recent study, researchers harnessed physiologic features that proved effective in classifying communication attempts (protest, demand, or comment) and behavioral states (pleasure, displeasure, or neutral) in individuals with PIMD, achieving accuracy rates exceeding 48% through ML algorithms (54). Moreover, although this has been proven futile, there have been endeavors to explore methods enabling individuals with PIMD to control physiological signals for interaction independent of motor movements (10). While we did not collect specific physiological data to support our findings, some physiological investigations may provide explanations and offer a more holistic understanding of the observed behaviors, potentially uncovering a comprehensive picture of the factors influencing the behavior outcomes among children with PIMD/IDs. Elevated atmospheric pressure has been linked to mood regulation through its influence on the metabolism of serotonin, a neurotransmitter associated with feelings of well-being and happiness (20, 55). This suggests that elevated or changes in atmospheric pressure may directly impact the brain’s serotonin levels, affecting an individual’s mood and emotional state. In the context of our study, it is possible that children exhibited more attentive and interested behaviors, indicating greater engagement with their caregivers, during pleasant and favorable weather conditions when atmospheric pressure is stable or elevated. The stabilizing or elevating effect on serotonin levels during such weather conditions could contribute to the observed positive changes in behavior, making the children more receptive and engaged during these times. We also consider that the relationship between high engagement behaviors during fall may be explained by the fluctuating levels of [3H]Paroxetine binding in platelets, which plays a role in mood regulation. A study found significantly lower [3H]Paroxetine binding in platelets during fall compared to winter, with a significant portion of the variance attributed to weather variables such as atmospheric pressure (56). This finding may provide evidence for the influence of atmospheric pressure on the increased engagement of children with PIMD/IDs during fall compared to winter. While it is unclear how the binding mechanism itself affects behaviors, previous research has suggested that individuals with depression exhibit lower levels of [3H]Paroxetine binding (57). Thus, it is plausible to assume that children with PIMD/IDs may have higher [3H]Paroxetine binding to platelets, which could contribute to their increased engagement. Although this study does not provide a direct explanation for this relationship, it sheds light on the potential relevance of considering [3H]Paroxetine binding, atmospheric pressure levels, and seasonal variations for future investigations to understand the behaviors of children with PIMD/IDs.

Undesirable weather conditions, marked by low-pressure levels and strong winds, notably led to more instances of children displaying refusal or disagreement behaviors. Low or fluctuating atmospheric pressure systems have demonstrated links to altered activity levels in children and an increased risk of migraine attacks, epileptic seizures, depressive symptoms, and cardiovascular complications in adults (12, 14, 25, 26, 58). A study has shown that the transient lowering of barometric pressure associated with the approach of low-pressure systems, like typhoons, can induce craniofacial sensations such as ear pressure, head compression, and sometimes mild headaches due to an imbalance in pressure between the external and middle ear, causing deformation and tension of the tympanon (59). This observation aligns with the concept that atmospheric pressure fluctuations triggering migraines can result from the dilation of cerebral blood vessels. This dilation leads to serotonin release from platelets, which, in turn, causes vasoconstriction and initiates an aura. Subsequently, a rapid decrease in serotonin levels results in the rapid dilation of cerebral blood vessels, ultimately triggering a migraine (25). While the exact mechanisms underlying these sensations in children with PIMD/IDs remain unclear, we propose a potential connection. We suggest that atmospheric pressure changes, particularly approaching or within low-pressure systems, may elicit sensations of ear pressure, head compression, and occasional mild headaches. These sensations could lead to discomfort, potentially impacting their overall well-being and mood (60). In children with PIMD/IDs, these sensory experiences may be expressed as refusal or disagreement behaviors. Nevertheless, the precise mechanisms of this connection, particularly in the context of children with PIMD/IDs, still need to be understood and necessitate further investigation.

During the winter months, increased cloud cover and the resultant reduction in daylight and natural light exposure have been observed to affect the attentional focus, concentration, and interest levels in children with PIMD/IDs. The connections between light exposure and mood or behavior are thought to be mediated through various biological mechanisms, including the modulation of circadian rhythms, activation of emotional brain regions, and melatonin production (22, 61, 62). For children with PIMD/IDs, who often have unique sensitivities and responses to environmental stimuli, alterations in their daily light exposure might have disrupted their circadian rhythms. Such disruptions can lead to changes in their mood and behavior, potentially causing fluctuations in their engagement, interest, and overall emotional states during winter. Although the precise mechanisms driving these effects are complex and warrant additional research, they highlight the significance of factoring in environmental elements, such as light exposure, particularly in the winter months with heightened cloud cover, when developing interventions and assistance for children with PIMD/IDs.

Moreover, the robust positive effect observed in our study, indicating that increasing outdoor humidity during winter positively influenced the assent behaviors of children with PIMD/IDs, suggests that these children exhibited more attuned agreement when exposed to a fair or comfortably humid environment, particularly during the cold seasons. This response could be attributed to the physiological effects of increased outdoor humidity, which include stimulating heightened blood flow to the skin’s surface. This increased blood flow facilitates heat dissipation through the evaporation of sweat, leading to elevated heart rates, respiratory ventilation rates, and mean skin and eardrum temperatures (63, 64). These physiological changes, brought about by higher humidity levels, may have contributed to an overall sense of comfort and well-being for children with PIMD/IDs. Consequently, these effects may explain their increased attunement during the winter, even indoors.

In contrast to the hypothesis regarding the potential random effects of children’s characteristics on behavior and emotional states, our findings revealed that the weather-related effects on the children’s behaviors and emotional states are not significantly mediated by individual characteristics such as age, gender, education level, and conditions. Despite the fact that children with PIMD/IDs possess unique and individualized movement and behavioral repertoires, the variability in their characteristics did not strongly influence their behavior outcomes. It is possible that fluctuations in weather indices, seasonal changes, and time of the day played a more prominent role in shaping their behaviors, resulting in comparable outcomes across individuals. Furthermore, the random effect of proximity sensing or location was also found to be insignificant in our study. This could be attributed to all participants being recruited from the same school, and the data collection sessions were confined to classroom settings. Therefore, the physical proximity or specific location within the school did not significantly impact the observed behavior patterns.

In order to ensure a comprehensive interpretation and generalization of the potential main and seasonal interaction effects of weather index fluctuations and time of the day as predictors of behavioral and emotional variations in children with PIMD/IDs, it is crucial to acknowledge several methodological limitations. One limitation lies in the data collection sessions’ relatively short duration and limited seasonal coverage. Although including 105 sessions can be considered a strength of this study, the sessions were relatively brief, with an average length of 18.5 minutes. Additionally, data were only collected during two seasons, which may not fully capture weather indices’ main and seasonal interaction effects on the observed outcomes. It is also essential to acknowledge the limitation arising from the relatively low number of data points on positive and emotional states, which led to insignificant findings. However, we also recognize that the slight temperature changes during the fall and winter seasons might not have significantly impacted the children’s emotions, in contrast to situations involving high temperatures, like in summer. Most importantly, although we focused on collecting data from interactions initiated by the children, it is important to acknowledge the potential influence of proximal variables such as the interaction context, the overall caregiver relationship, or the specific situation or task. These variables may have introduced confounding factors that falsely attribute effects to distal variables such as indoor and outdoor weather indices, even when there may not be any direct relationship between them. By acknowledging these methodological limitations, we can better contextualize and interpret the results, allowing for a more nuanced understanding of weather indices’ main and seasonal interaction effects on the behaviors and emotional states of children with PIMD/IDs.

We also consider our approach to interpreting behavioral observations in a physiological context as a limitation. Once more, we stress that although we did not collect physiological data, the integration of physiological explanations can provide a more comprehensive understanding of the behaviors observed. This approach may unveil a fuller picture of the factors influencing the outcomes among children with PIMD/IDs. It is important to highlight that presenting potential physiological explanations not only serves as a means to inspire hypotheses but also as a catalyst for more in-depth investigations into the potential mechanisms and pathways by which environmental factors like weather indices and seasonal variations may impact the functioning of the autonomic nervous system and, consequently, influence the behaviors of children with PIMD/IDs. As we move forward, we actively explore integrating physiological data into our innovative computer-based communication-aid assistive technology (AT) system, rooted in an ML framework. This expanded system, in addition to tracking observed movements, has the potential to incorporate physiological data, thus enhancing our comprehension of the intricate interplay between physiological factors and behavior in children with PIMD/IDs.

Overall, this study provides compelling evidence that fluctuations in weather indices, including seasonal changes and time of the day, can provide potential pathway indicators and supplement behavioral observations to elicit the behavioral states of children with PIMD/IDs. The results indicate that outdoor weather variables and seasonal variations in atmospheric pressure, cloudiness, wind speed, and humidity are robust predictors of behaviors, surpassing the impact of indoor indices. These outdoor weather indices and seasonal changes are likely linked to variations in physiological signals and functions, influencing the occurrence and changes in behaviors among these children. The implications of these findings are significant for caregivers, teachers, and parents who interact with children with PIMD/IDs. Awareness and sensitivity to the levels and fluctuations in weather indices and seasons are crucial in understanding and catering to the needs and preferences of these children. For instance, it is advisable to encourage outdoor activities during fair or pleasant weather to promote social interaction and communication skills. During cloudy and windy winter days, in addition to providing a warm and humid indoor environment, caregivers should engage children in stimulating indoor activities that foster attentiveness, concentration, and sustained motivation, particularly during recess or breaks, to mitigate the likelihood of refusal or disagreement behaviors. Caregivers can adapt their routines on hot and humid afternoons to keep children cool and comfortable. This might involve ensuring air conditioning, using fans, or providing cool sensory experiences to prevent irritability and discomfort. On warm and sunny mornings during the fall, activities can be planned to take advantage of the positive emotional state of the children, perhaps with outdoor time or engaging sensory experiences. Recognizing the impact of weather conditions on sensory experiences, educators can design sensory-friendly environments. For example, when some children might become more nervous during rainy or stormy weather, educators can create calming spaces with soft lighting, soothing sounds, and comforting textures to ease anxiety. In contrast, they can provide stimulating sensory activities on sunny days to harness the enthusiasm and emotional strength associated with such conditions. In response to the effects of weather on behaviors, age-appropriate recreational and extracurricular activities can be thoughtfully planned. For instance, during colder seasons, educators can schedule indoor activities that keep children engaged and active, combating the reduced negative affect and increasing alertness associated with such weather. On rainy days, children can enjoy creative indoor art projects to channel their energy positively. Educators can adjust classroom arrangements and schedules to create a supportive and conducive learning atmosphere. During seasons with weather-related discomfort, such as hot summers, educators can ensure that classrooms remain cool and well-ventilated. Conversely, during winter, when increased sunshine is associated with positive emotional states, classrooms can maximize natural light to enhance alertness and reduce negative affect. These examples illustrate how tailored strategies, influenced by weather-related insights, can enhance the lives of children with PIMD/IDs by providing them with more comfortable, positive, and enriching daily experiences.

## Data availability statement

The original contributions presented in the study are included in the article/supplementary material, further inquiries can be directed to the corresponding author.

## Ethics statement

This study involving humans was approved by the ethics board of the Faculty of Education, Ehime University. The study was conducted in accordance with international ethical guidelines and regulations. Written informed consent for participation in this study was provided by the participants’ legal guardians/next of kin.

## Author contributions

VH: validation, formal analysis, writing-original draft and review and editing, and visualization. TK: conceptualization, methodology, funding acquisition, project administration, supervision, writing-review and editing, and resources. AT: validation, formal analysis, data curation, and writing-review and editing. YF: methodology, validation, formal analysis, investigation, data curation, and writing-review and editing. SS, EO, and TS: conceptualization, software, resources, project administration, and writing-review and editing.
